# *OnDA*: online data analysis and feedback for serial X-ray imaging^1^

**DOI:** 10.1107/S1600576716007469

**Published:** 2016-05-23

**Authors:** Valerio Mariani, Andrew Morgan, Chun Hong Yoon, Thomas J. Lane, Thomas A. White, Christopher O’Grady, Manuela Kuhn, Steve Aplin, Jason Koglin, Anton Barty, Henry N. Chapman

**Affiliations:** a Centre for Free Electron Laser Science, Deutsches Elektronen Synchrotron DESY, Notkestrasse 85, 22607 Hamburg, Germany; b Deutsches Elektronen Synchrotron DESY, Notkestrasse 85, 22607 Hamburg, Germany; c European XFEL GmbH, Albert Einstein Ring 19, 22761 Hamburg, Germany; d Linac Coherent Light Source, SLAC National Accelerator Laboratory, 2575 Sand Hill Road, Menlo Park, CA 94025, USA; e Department of Physics, University of Hamburg, Luruper Chaussee 149, 22761 Hamburg, Germany; f Centre for Ultrafast Imaging, Luruper Chaussee 149, 22761 Hamburg, Germany

**Keywords:** online data analysis, free-electron lasers, serial femtosecond crystallography, computer programs

## Abstract

This article describes a free and open-source data analysis utility designed for fast online feedback during serial X-ray diffraction and scattering experiments: *OnDA* (online data analysis). Three complete real-time monitors for common types of serial X-ray imaging experiments are presented. These monitors are capable of providing the essential information required for quick decision making in the face of extreme rates of data collection. In addition, a set of modules, functions and algorithms that allow developers to modify the provided monitors or develop new ones are provided. The emphasis here is on simple, modular and scalable code that is based on open-source libraries and protocols. *OnDA* monitors have already proven to be invaluable tools in several experiments, especially for scoring and monitoring of diffraction data during serial crystallography experiments at both free-electron laser and synchrotron facilities. It is felt that in the future the kind of fast feedback that *OnDA* monitors provide will help researchers to deal with the expected very high throughput data flow at next-generation facilities such as the European X-ray free-electron laser.

## Abstract 2

This article describes the software package *OnDA*: online data analysis and feedback for serial X-ray imaging.

## 1. Introduction   

The advent of X-ray free-electron lasers (XFELs) has opened new possibilities for structural biology (Spence *et al.*, 2012 ▸). The ability of XFELs to deliver a very high radiation dose to the sample within femtosecond time scales has been exploited in several newly developed techniques, mostly based on the so-called ‘diffraction-before-destruction’ method (Neutze *et al.*, 2000 ▸). Some of the novel approaches involve imaging of small protein crystals (serial femtosecond crystallography; SFX) (Chapman *et al.*, 2011 ▸) and pump–probe experiments (Aquila *et al.*, 2012 ▸; Kern *et al.*, 2013 ▸; Spence *et al.*, 2012 ▸; Neutze & Moffat, 2012 ▸). In other experiments, single mol­ecules or viruses are imaged, in isolation (Seibert *et al.*, 2011 ▸; Bogan *et al.*, 2008 ▸; Saldin *et al.*, 2011 ▸) or aligned in an electromagnetic field (Kierspel *et al.*, 2015 ▸).

In most of these techniques, samples are constantly flowed in a liquid or gaseous jet across a pulsed X-ray source which has a repetition rate of up to 120 Hz. Significant amounts of sample are consumed in a very short time, and the data generated by the instruments requires a large amount of storage space. Furthermore, experimental parameters, such as the degree of molecular alignment in controlled imaging experiments, or the hit rate and resolution in an SFX experiment, must be kept within acceptable bounds. By monitoring experimental conditions in close to real time, the experiment may be maintained in optimal alignment, or alternatively, one may pause the experiment to correct unfavorable conditions, thereby preventing the collection of unfavorable data while preserving valuable sample.

Future instruments under development will feature even higher pulse repetition rates in excess of 1000 frames per second, making it impractical for facilities to store all of the recorded data. Thus real-time analysis and data reduction will become a necessity. For example, the European XFEL, scheduled to commence operation in 2017, will be capable of recording diffraction data using 27 000 pulses per second (Altarelli, 2006 ▸), while the LCLS-2 project foresees megahertz pulse repetition rates.

Some software packages for real-time monitoring of XFEL experiments have been developed in recent years. The *CASS* program (Foucar *et al.*, 2012 ▸) has been created specifically for this purpose. *Cheetah* (Barty *et al.*, 2014 ▸) was originally developed for high-throughput offline data reduction, but can be run at the Linac Coherent Light Source (LCLS) as a module of the data-processing framework *psana* (Damiani *et al.*, 2016 ▸) for real-time data analysis. *Cctbx.xfel* can also be used to analyze data as they are collected using the *Data Exploration Toolkit* (Sauter *et al.*, 2013 ▸; Zeldin *et al.*, 2015 ▸). All these packages, however, depend on either multi-thread or multi-process technology for parallelization, which limits them to running on a single computer. Some effort will be needed to adapt them for the quantity of data expected from future instruments. The recently released *Hummingbird* (Daurer *et al.*, 2016 ▸), initially developed for single-particle imaging at LCLS, is, to our knowledge, the only framework that is designed to scale beyond a single machine.

We introduce *OnDA*, a new software framework for real-time monitoring of X-ray imaging experiment data and experimental conditions. The *OnDA* project aims to provide users with a set of stable and efficient real-time monitors for the most common types of experiments. These can be used immediately without modifications or can be easily adapted to meet the users’ requirements. In addition, we provide a set of modules to easily build real-time monitoring programs tailored to characteristics of specific experiments. *OnDA* processes imaging data in the broadest sense: multidimensional and multiple-pixel data (for example, a diffraction pattern or a photoemission spectrum, but also an image coming from a camera or a microscope), but also any kind of digital output from an instrument or sensor (for example, temperature readout, beam and pulse, energies). The *OnDA* project focuses on scalability and portability, to facilitate its adoption for a wide array of current and future instruments, and also strives for stability and performance. In order to achieve these goals, the *OnDA* framework implements a master/worker parallelization paradigm using the Python interpreted scripting language (http://www.python.org) and relying on free and open-source libraries and protocols. These libraries are available on all of the most widely used computer platforms and architectures and have been actively used in large-scale deployments in many scientific environments. The use of the Python programming language, which is particularly suited to prototyping and rapid development, makes *OnDA* easy to modify and to adapt to the requirements of specific experiments, especially when these requirements emerge ‘in the field’ during the course of the experiment itself. The *OnDA* project also aims to keep the code base simple and as small as possible. The focus is on providing a core set of functions, while allowing the framework to be expanded with external software when possible, avoiding the need to reimplement already optimized algorithms.

In the following sections we briefly outline *OnDA*’s architecture and introduce the set of real-time monitors that are currently distributed with the framework. We then briefly discuss the backends required to integrate *OnDA* into the software frameworks of two large-scale facilities. Finally we describe how to obtain *OnDA*’s source code and the focus of future development.

## 2. *OnDA* framework architecture   

**Figure 1:**
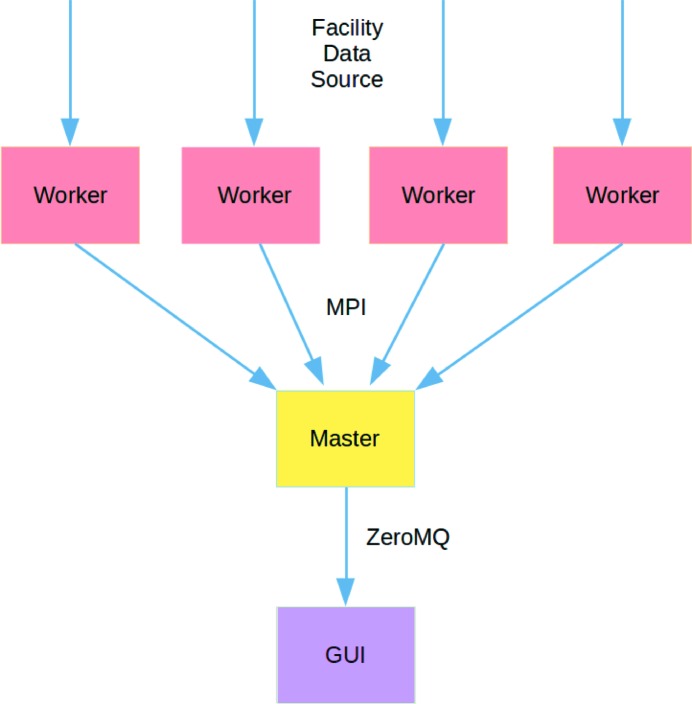
The *OnDA* framework is built using a master/worker architecture. The workers recover and process the data, the result of the processing is sent to the master for further processing, and eventually dispatched to the graphical user interface (GUI). The workers and the master communicate with each other using the MPI protocol, while the master and GUI communicate using the ZeroMQ protocol.

The *OnDA* framework is based on a master/worker architecture (see Fig. 1 ▸) and operates on data provided by an experimental facility’s data acquisition system (DAQ). The DAQ software, running within a facility’s software framework, collects concurrent readings from a wide range of instruments and detectors and groups them in a collections of time-related readouts, called ‘events’. Each event corresponds to the instrument state and measurements for a single pulse from an X-ray free-electron laser, or a single detector readout at a synchrotron source.

Worker nodes retrieve single-event data from a source, extract the important information and carry out any required processing. The resulting data are then sent to the master node, which can carry out further multi-event processing (for example, aggregation of data from many workers or averaging) and can optionally send information to the graphical user interface (GUI) to be displayed. Worker nodes communicate between themselves and with the master node using the MPI framework (http://www.mpi-forum.org), while data are transferred from the master node to the GUI using the ZeroMQ (ZMQ) protocol (http://zeromq.org). In *OnDA*, a single worker of the master node runs on a single CPU core.

**Figure 2:**
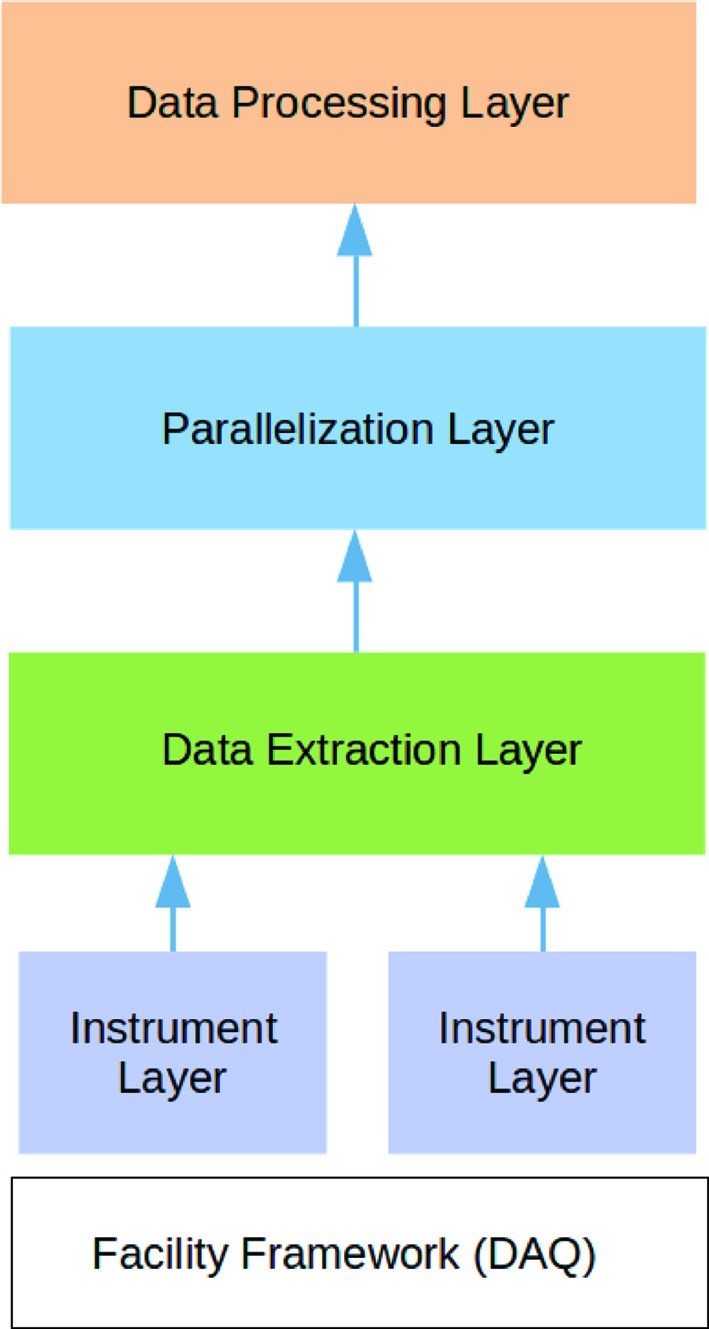
The *OnDA* framework features four distinct layers, as described in the text. In the diagram, data flow from the bottom to the top, from the facility framework which provides event data to the Data Processing Layer that processes the data and prepares it for visualization.

*OnDA* is highly modular. The functions that carry out the scientific data processing are clearly separated from the functions that carry out data retrieval, data extraction and communication between the nodes. The *OnDA* framework groups these functions into four layers (see Fig. 2 ▸).

(i) Data Processing Layer: this layer carries out the scientific data processing. *OnDA* implements in this layer the specific functions that perform calculations on the data (for example, peak extraction or background subtraction). These functions can run both on the worker and on the master nodes. The nature and capabilities of each type of real-time monitor are essentially defined by the functions implemented in this layer, so its content is likely to be unique for each type of experiment.

(ii) Parallelization Layer: the Parallelization Layer takes care of the communication between the worker and the master node, and between different worker nodes. Furthermore, this layer contains functions used by the worker nodes to recover event data from a data source. Since different data sources require different data retrieval strategies, the functions that are implemented in this layer strongly depend on the nature of the data source (files, shared-memory server *etc*.).

(iii) Data Extraction Layer: this layer implements the functions that extract relevant information from event data (for example, a detector’s recorded image, or the numerical value corresponding to a sensor’s output). The functions implemented in this layer depend strongly on the format of the event data. Each real-time monitor imports the implementation of the Data Extraction Layer required by the data format.

(iv) Instrument Layer: this layer implements functions for data extraction that are specific to a single instrument, detector or sensor. Irrespective of the data format, it is often necessary to know some details about the way a specific instrument encodes information: for example, which channel of a multi-channel instrument contains the required value. The Data Extraction Layer includes units from this layer depending on the instruments used for a specific experiment.

The implementation details of the Parallelization, Data Extraction and Instrument Layers strongly depend on the software framework on which the DAQ providing the data runs, and on the format of the provided data. Hence, we collectively label these layers ‘backend’. The backend exposes data to the Processing Layer (which we label ‘frontend’) in a standardized facility-independent format, providing a clear separation between facilty-dependent and -independent code in the *OnDA* framework.

The following paragraphs contain a brief descripition of the data flow through a typical real-time monitor created using the *OnDA* framework. Courier font is used in the text to refer to the names of classes, functions or data structures.

**Figure 3:**
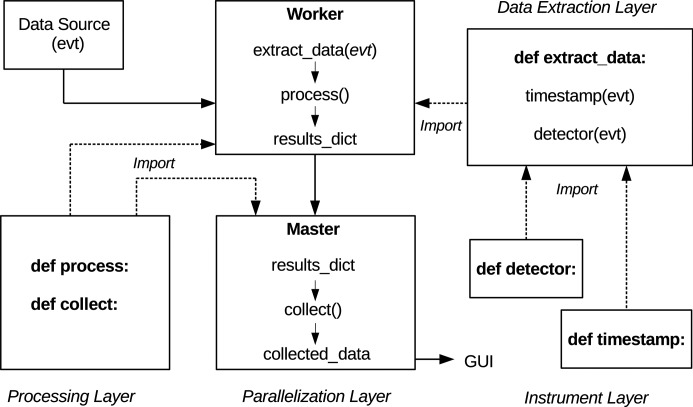
Data workflow in *OnDA*. Each worker node collects event data from a source, which can be a shared memory server, a file or a network data stream. It then calls the extract_data function, which is imported from the Data Extraction Layer, to recover useful information from the event data (a detector readout, an instrument reading) and makes it available as a class property. The worker then calls the process function, imported from the Processing Layer, to process the information, and stores the results in a Python dictionary called results_dict. This dictionary is then sent to the master node using the MPI framework. Upon receiving the data, the master note calls the collect function, which carries out further processing, and stores what needs to be sent to the GUI in another Python dictionary called collected_data.

A real-time monitor is implemented as the onda Python class which is defined in the Parallelization Layer. This class is instantiated on all nodes and makes sure that each node is correctly initialized according to its role. Any required function defined in one of the other layers is imported during the initialization. Worker nodes import the extract_data function defined in the Data Extraction Layer, which in turn imports all the required instrument-specific functions defined in the Instrument Layer. Workers also import the process function from the Data Processing layer, while the master node imports the collect function from the same layer. A real-time monitor hence contains, through a chain of inclusions, functions from all four layers. Fig. 3 ▸ shows a typical monitor implementation, and traces the flow of data through the master and worker nodes.

Each worker node fetches event data from the event data source and calls the extract_data function. This function extracts the required information from the event data and stores it in the form of properties of the onda class. The name and the content of the properties depend on the specific extract_data function and are described in the documentation of the relevant function. For example, the timestamp of the event data is typically stored in a property called self.event_timestamp, the raw detector readout is stored in a property called self.raw_data, and so on. After the information has been extracted, the worker nodes call the process function, which carries out the data processing (for example, background correction and detection of diffraction peaks). This function operates on the class properties and stores any information that needs to be sent to the master node in a Python dictionary called results_dict.

After receiving data from a worker, the master node stores them in a property called results_dict and calls the collect function. This function is called on the master node every time data are received from one of the workers and performs data processing on the aggregated data from multiple events. There is no default strategy for data aggregation: the preferred approach can be implemented by the developers of each real-time monitor in the collect function. After the function finishes running, the content of the results_dict dictionary is discarded. It is replaced by new data coming from other worker nodes when the function is called again. The master node stores by default no information between function calls, unless this functionality is implemented in the collect function. The required information is finally packaged in a dictionary called collected_data and sent to the GUI for visualization.

The core scientific data processing is carried out by the process and collect functions. They can make use of Algorithms to carry out their task. Algorithms are implemented using Python classes and are instantiated on both the worker and the master nodes at startup with a common set of initial parameters. They can be invoked (using the apply method) from both types of nodes, and perform different tasks depending on the identity of the caller. Algorithms allow developers to implement full data processing pipelines, spanning across both worker and master nodes, in a single Python object, resulting in clearer code that is easier to read, modify and reuse.

Algorithms are generally implemented using Python, augmented with the *NumPy* (http://www.numpy.org) and *SciPy* (http://www.scipy.org/) libraries. For some complex algorithms, however, the processing speed of an interpreted programming language is not sufficient. For these algorithms *OnDA* uses compiled languages (like C or C++). A thin Python wrapper is then written for them using the Cython (http://cython.org/) Python extension. Wrappers can also expose the function calls of the external programs to *OnDA*, providing two-way conversion of data formats as well as input and output parameters.

The GUI is implemented using the *Qt* development toolkit (http://www.qt.io) and the fast-display *PyQtGraph* library for scientific graphics (http://www.pyqtgraph.org). The communication between the master node of the monitor and the GUI takes place over a network connection using the ZeroMQ protocol, which relies on the TCP network communication protocol. Therefore, a network connection with at least one open TCP port is required for the connection. While the ZeroMQ protocol is not as fast as the MPI protocol used by the nodes to communicate between themselves, its reliance on TCP allows communication across sub-networks and even across the internet. *OnDA* exploits the speed and flexibility of MPI for interprocess communication between worker and master processes performing the scientific computing, while broadcasting results to one or more GUI displays over ZMQ enables the GUI clients to be run independently on hosts located remotely on different networks. We have found this to enhance stability and fault tolerance because the GUI network can suffer from a delay, or the GUI can even terminate, without affecting the *OnDA* analysis processes. Many instances of the GUI, running on several different machines, even outside of the facility where the experiment takes place, can connect to the same monitor at the same time. However, the monitor will continue to run even with no GUI attached, enabling it survive faults such as network dropouts or the user accidentally closing the GUI.

The *OnDA* framework is also designed to be strongly resilient to errors and missing data, and to survive a crash of one or more working nodes. If the facility framework fails to provide data to one or more workers, the affected nodes simply wait for data while the other nodes keep processing. The framework also checks data for errors. If the recovered event data set is incomplete or corrupted, workers simply skip the corrupted data and move to the next event. Furthermore, if an unexpected condition causes an unrecoverable crash of one or more workers, the others will continue processing events and the monitor will keep working, although with a reduced performance, as long as the master node is running. In very high throughput experiments, or when an *OnDA* monitor is running on limited computer resources, it is possible that the speed at which the DAQ provides data will be too high for the monitor to process an event before the next one is received. In these circumstances the *OnDA* framework skips processing the oldest events, guaranteeing that the processed ones are as recent as possible.

It should be pointed out that, although all the real-time monitors currently distributed with the *OnDA* framework are implemented using a single master node and multiple workers, the framework’s architecture does not prevent the development of monitors featuring multiple master nodes with different roles, or even multiple layers of master nodes. This flexibility, achieved by implementing inter-node communication using the MPI protocol, markedly increases scalability.

## 3. Available real-time monitors   

The *OnDA* project provides, at this time, three complete real-time monitors: a monitor for SFX, a monitor for velocity map imaging (VMI) and a monitor for serial fiber diffraction imaging. The three monitors are described in detail in the following sections and can be run using the backends described later as they are provided, without further modification. However, all modules, functions and algorithms that were used to create them (for example, peak finding algorithms, functions that perform detector corrections, averaging and input/output routines) are available as separate entities in the *OnDA* framework. By combining them in different ways, developers can easily create variations on the provided monitors, or completely new ones designed for different experiments.

### 3.1. SFX monitor   

The SFX monitor allows users running an SFX experiment to keep hit rate (the fraction of X-ray shots that resulted in a hit) and saturation rate (the fraction of shots where more than three of the detected peaks appeared to have reached the saturation threshold of the detector) under control. Furthermore, it uses the collected data to display a constantly updating virtual powder pattern. The monitor reads the detector data, applies detector corrections and searches for Bragg peaks in the resulting data. It can optionally apply a mask or carry out gain correction according to a gain map provided by the user. The *peakfinder* algorithm from the *Cheetah* program is used to detect potential Bragg peaks. The user can alter the sensitivity of the algorithm using all the options and parameters available in *Cheetah*. On the basis of the number of peaks found, the monitor decides if the detector frame represents a diffraction pattern and updates the hit rate accordingly. The intensity of each Bragg peak is also checked against a user-provided threshold to see if it is outside of the dynamic range of the detector. The collected data are then sent to the GUI for visualization. All options are configured using a plain-text configuration file.

**Figure 4:**
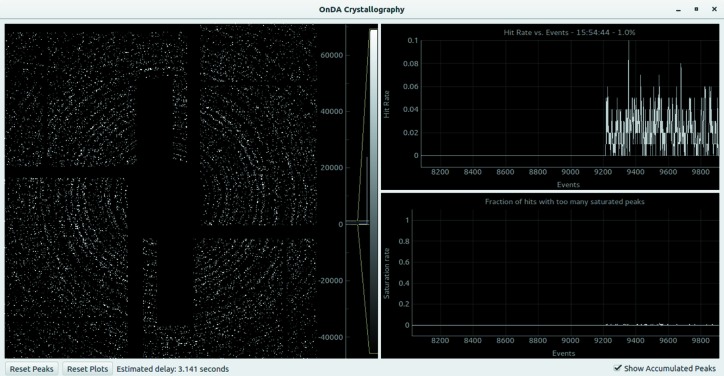
Graphical user interface for the *OnDA* crystallography monitor. The left side of the screen shows a virtual powder representation of the incoming data, while the right side shows a running average of the hit rate and of the saturation rate (see main text for definitions of these rates). The estimated delay between the time of data collection and of data display is reported below the main panels.

The GUI of the SFX monitor (see Fig. 4 ▸) displays on the left a virtual powder pattern generated from the detected Bragg peaks. The right side is occupied by two plots showing the recent history of the running averages of the hit rate (at the top) and of the saturation rate (at the bottom). The most recent values of the running averages lie on the right-hand side of the plots. The size of the time-point window used to compute the running averages can be chosen by the user. By clicking on the plots, specific time points can be marked with a vertical line. A right click on the line removes it. A time point corresponding to changes of important experimental conditions can be marked using this feature, resulting in clearer visual feedback on the effect of the change. Buttons at the bottom of the main window allow the user to clear both the virtual powder pattern and the plots. An estimate of the delay between the time when the data are collected and when the result of the processing is displayed in the GUI is also reported.

We routinely use the SFX monitor for our experiments at the CXI instrument at the LCLS facility, located at the SLAC National Accelerator Laboratory in Menlo Park, CA, USA. We run it on the same machines where the DAQ is running, to allow memory sharing and direct communication between the DAQ and *OnDA* without passing through the filesystem. The DAQ at the CXI instrument uses five machines, which feature an Intel Xeon E5620 CPU with eight cores, a clock speed of 2.40 GHz and 48 GB of RAM, to process collected data in parallel, so each of the machines gets 20% of the event data. We found that running the SFX monitor with just four workers per machine, each running on a single logical core of the CPU (20 workers in total plus the master running on a total of 21 CPU cores), allows us to process all event data coming from the DAQ without losing any data. Each *OnDA* worker uses around 2 GB of virtual memory, while the master uses around 750 MB of memory. The delay between data collection and visualization in the graphical interface is usually between 1 and 2 s under typical network load conditions.

The SFX monitor has been used also for experiments at the P11 bio-imaging and diffraction beamline of the PETRA III synchrotron located at DESY in Hamburg, Germany. The monitor was running on a machine with an Intel Xeon E5-2650 CPU featuring a clock speed of 2.60 GHz and 32 cores. Running *OnDA* with just 16 workers (plus a master node) allowed us to process all of the data, with a delay between data collection and visualization of about 4–5 s, using a total of 17 CPU cores.

### 3.2. VMI monitor   

This monitor was designed to measure the degree of laser-induced alignment of particles in a molecular beam using the VMI technique (Eppink & Parker, 1997 ▸). It recovers the data from a CCD camera imaging the phosphor screen of a VMI spectrometer and applies a simple peak detection algorithm to detect the ion signal. Each signal peak is also located with sub-pixel precision using a center-of-mass centroiding algorithm. Data from a predefined number of events (chosen by the user) are accumulated. Some statistics that estimate the degree of alignment of the ions with respect to the alignment laser (vertical axis of the VMI spectrometer) are computed. Since the center of the spectrometer screen is often displaced with respect to the experimental interaction point, the user can apply translational and rotation offsets to the data before the statistics are computed. The data are then sent to the GUI for visualization.

**Figure 5:**
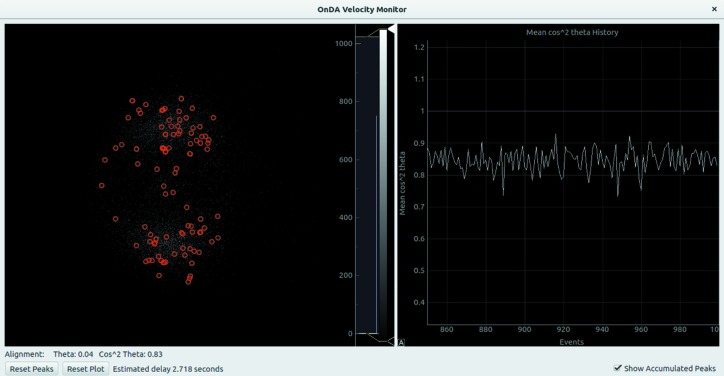
Graphical user interface for the *OnDA* velocity monitor. The screen shows on the left the signal peaks detected on the VMI screen for the latest predetermined number of events. Below the main plot, the degree of alignment of the data along the vertical axis of the VMI screen is reported as values of θ and 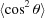. Only the data included in a user-defined wedged-shaped area of the screen are used to compute the alignment statistics. Behind the signal peaks, shown as red circles, the user can choose to show the wedge-shaped area, or, like in the above screenshot, the accumulated peak density. On the right side of the screen, the evolution of the 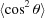 alignment statistic over time is shown.

The GUI of the velocity monitor (see Fig. 5 ▸) shows the detected signal peaks as red circles. The user must define (though a configuration file) a wedge-shaped area on the VMI screen. Only peaks within the area are included in the statistical calculations. The area is shown in white in the GUI. By checking a tick box, the user can display an accumulation plot of the detected signal peaks in its place. Below the main viewer, the degree of alignment statistics θ and 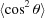 are shown, and under those, an estimation of the delay between data collection and display. The right-hand side of the monitor displays the recent history of the 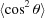 alignment statistic.

### 3.3. Serial fiber diffraction monitor   

This monitor was developed for a fiber diffraction experiment at the LCLS. In this experiment individual fiber bundles were injected into the path of the FEL beam *via* a liquid jet. Pressure differentials in the liquid jet align each fiber bundle along its long axis to that of the jet, referred to as ‘flow alignment’. The axis of alignment could then be scanned by tilting the liquid jet with respect to the beam axis, allowing fibers to be imaged over a large range of orientations. The collected diffraction data were subsequently computationally aligned to form a view of the reciprocal space volume of the fibers.

The serial fiber diffraction monitor was designed to observe the hit rate of the fibers as well as the background signal from the liquid jet in real time.

**Figure 6:**
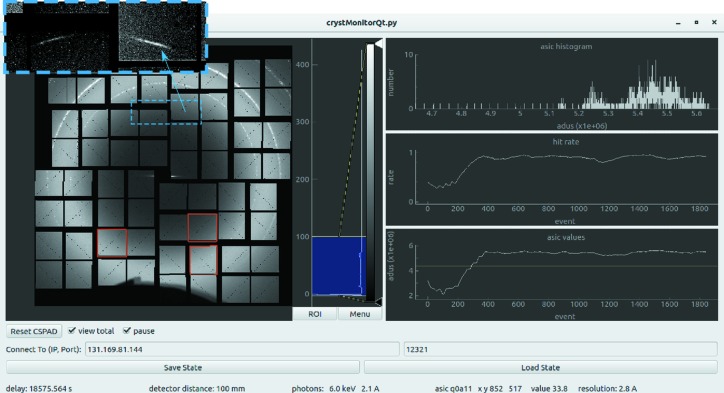
Snapshot of the *OnDa* graphical frontend used to monitor serial fiber diffraction collection at the LCLS CXI end station. (Left panel) Live view of individual or integrated diffraction patterns as recorded by the CSPAD detector. (Inset top left) Outlined in blue, fiber bundle diffraction peaks as seen with radial background subtraction turned on. (Top right panel) Histogram of the integrated signal on the selected asics (CSPAD detector panels) as outlined in red in the left panel. (Middle right panel) Time-averaged hit rate. (Lower right panel) Integrated signal of the selected asics as a function of time. (Bottom panel) User input options for the GUI in addition to live output of scalar data such as the detector distance, the photon energy, and the detector counts and resolution of the pixel under the mouse.

Fig. 6 ▸ shows a snapshot of the *OnDA* GUI used during the experiment. In the left panel we see the integrated diffraction intensity over approximately 1000 detector frames. Here we can see the background due to scattering from the liquid jet nozzle as arcs across the top of the image, as well as a diffuse background ring from the liquid buffer. The integrated signal on each detector panel was stored for each shot by *OnDA*. These were sent to the GUI and could be observed simply by clicking on the desired panels, which are then outlined in red as shown. This allows the user to monitor the signal level on the detector arising from different sources, by selecting a panel most exposed to photons scattered by the object of interest. For example, the liquid jet was kept in the FEL focus by adjusting the nozzle position so as to maximize the background from the water ring, ensuring that the FEL beam passed through the center of the water column.

Diffraction from individual fiber bundles could only be seen after subtracting the water background. We chose a simple *OnDA* module that performs radial background subtraction for each data frame before it is sent to the GUI (this was followed by a more detailed treatment of the data offline). To achieve this we subtract the mean of the signal in each resolution ring after correcting for the azimuthal modulation in the diffraction intensity due to the polarization axis of the FEL beam. The result is shown in the top left inset of Fig. 6 ▸, outlined in the blue dashed box, where two diffraction streaks from the fibers are visible.

## 4. Available backends   

The nature and characteristics of the data source strongly depend on the environment in which *OnDA* is running. When the user is running *OnDA* during an experiment, the software infrastructure of the facility is responsible for providing event data. When *OnDA* is run on a laptop or on a desktop workstation, files can be used as the data source. In addition to the nature of the data source, the instruments used during an experiment (detectors, cameras, digitizers) dictate the data extraction procedure. All this is implemented in the Parallelization, Data Extraction and Instrument Layers (collectively called the ‘backend’). The *OnDA* project currently provides three backends: the *psana* backend, the Petra III P11 backend and the filelist backend.

### 4.1. *Psana* backend   

The *psana* backend allows *OnDA* to be used at the CXI, XPP and AMO instruments at the LCLS facility. This backend allows *OnDA* to interface with the *psana* software framework in use at the facility. *Psana* can act as a data source both online (providing data to *OnDA* as they are collected for real-time monitoring) and offline (replaying a past experiment by streaming saved files). When *OnDA* is run as a real-time online monitor, it relies on shared memory monitors managed by *psana* to access the memory content of the machines that collect the data. When *OnDA* is run as an offline non-real-time monitor, it reads the data stream that *psana* generates from the saved files. The *psana* backend can be used with all three monitors (crystallography, velocity imaging and fiber diffraction).

### 4.2. Petra III P11 backend   

The Petra III P11 backend allows *OnDA* to be used at the P11 beamline of the PETRA III facility. This backend receives a data stream over a ZMQ connection from a data provider, which is implemented within the facility software framework. As soon as the detector software writes the data files, they are read and streamed to *OnDA* by the data provider. *OnDA* does not need to be run within the facility’s software framework as long as the machine where it is running can connect over the local network to the data provider. The Petra III P11 backend has, until now, only been used in serial crystallography experiments.

### 4.3. Filelist backend   

The filelist backend allows *OnDA* to rely on data files as the source of event data. *OnDA* scans a user-provided list of files, splits the list of files evenly amongst the worker nodes and processes each file. This backend can be used when the facility’s software framework does not provide access to a real-time data flow, but only allows users to access files saved in a filesystem. Files are often made available with some delay with respect to data collection, and monitors that rely on this backend often cannot provide real-time feedback. However, *OnDA*’s parallelized processing capabilities can still be used to very quickly analyze the data as soon as they are made available. If the delay is not too long, *OnDA* can still be used to provide quasi-real-time feedback.

The filelist backend can be used with all the monitors when no other data source is available. Additionally, it is useful to test the Processing Layer before the final deployment of *OnDA*. Currently, the Filelist backend supports CXI-format files (Maia, 2012 ▸), generic HDF5 files (The HDF Group, 2015 ▸) and CBF-format files (Bernstein & Hammersley, 2006 ▸).

## 5. Future developments   

To date we have used *OnDA* in more than ten experiments for live feedback. Roughly half of these were SFX experiments. Using this experience, we are currently developing new functionality for *OnDA* with a bias towards improving crystallographic applications. These new functions include the following:

(*a*) Online indexing of crystallographic Bragg peaks. This will be made possible by creating Python wrappers for indexing routines in *CrystFEL* (White *et al.*, 2012 ▸). As *CrystFEL* can index peaks found from a single detector frame, this process will be highly parallel and thus scalable in OnDA’s MPI framework.

(*b*) Improved crystal detection from found peaks. Currently *OnDA* determines that a detector frame contains diffraction from a crystal when the number of found peaks is above a threshold level. This can fail when too many peaks are found from background sources, such as hot pixels, ice rings or the tails of the liquid jet, requiring the user to increase the threshold. To overcome this problem we are developing a computationally ‘light’ algorithm that looks for crystallographic order in the found peaks without the need to execute expensive indexing routines to filter the data.

(*c*) Live configuration editor. We have often found, especially during the early stages of an experiment, that it is necessary to adjust or validate various parameters in *OnDA*, for example the peak finding parameters in an SFX experiments. How accurate the starting parameters need to be and how often the parameters need to be updated typically depends on the nature of the algorithms used by a specific monitor. For example, the current peak finding algorithm uses *Cheetah*’s *peakfinder8* algorithm (Barty *et al.*, 2014 ▸), and parameters typically need to be adjusted only when some important experimental condition changes (for example, the sample, the type of injector nozzle or the buffer used to dissolve the sample). Currently, optimizing these parameters requires running *OnDA* with different starting parameters and evaluating its performance, which in turn requires access to the machines running the worker nodes. However, we will make available a live configuration GUI, designed to allow the user to adjust parameters and preview their effect before writing them to the main configuration file and running *OnDA*.

(*d*) Pre-installation of *OnDA* in more facilities. As described, *OnDA* is currently configured to run, without any modification to the source code, for some beamlines in the LCLS and PETRA III facilities. We aim to make *OnDA* more easily available to users by continuing this work at other beamlines and facilities.

(*e*) Offline processing. The filelist backend allows *OnDA* to use files as the data source. Programs developed with the *OnDA* framework can then be run on saved data after the experiment is over. This opens the framework to the development of offline processing software that can take advantage of *OnDA*’s parallelization capabilities, and of *OnDA*’s algorithm and function libraries. We will add useful features for offline processing to the framework and we will develop programs for common processing requirements.

## 6. Access to *OnDA*   

The source code of the current stable version of *OnDA* can be downloaded from http://www.desy.de/~vmariani/onda. Since the *OnDA* framework is written in an interpreted language, no compilation is needed. Some algorithms, however, are implemented using the C and C++ programming languages and must be compiled if they are used by a real-time monitor created from the framework. Instructions for compilation are provided in the README.md file shipped with the *OnDA* source code. The source code for the development version of *OnDA* can be accessed by anyone using the git version control system at the following repository: https://stash.desy.de/scm/onda/onda.git. *OnDA* is distributed under the terms of the GNU General Public License, version 3 (https://www.gnu.org/licenses/gpl.html).

The *OnDA* framework can be used with the most recent version of Python 2 (2.7). It also requires the following Python modules to be installed in the system: *NumPy*, *SciPy*, *PyQt* (https://riverbankcomputing.com/software/pyqt/intro), *h5py* (http://www.h5py.org/), *mpi4py* (Dalcin *et al.*, 2011 ▸).

The Petra III P11 backend needs the *FabIO* python module (Knudsen *et al.*, 2013 ▸) to be installed. Furthermore, the *pyqtgraph* module is needed by the machine running the GUI.
